# Association between arterial stiffness and the presence of cerebral small vessel disease markers

**DOI:** 10.1002/brb3.1935

**Published:** 2020-11-19

**Authors:** Jae‐Han Bae, Jeong‐Min Kim, Kwang‐Yeol Park, Su‐Hyun Han

**Affiliations:** ^1^ Department of Neurology Chung‐Ang University Hospital Chung‐Ang University College of Medicine Seoul Korea; ^2^ Department of Neurology Seoul National University Hospital Seoul Korea

**Keywords:** arterial stiffness, enlarged perivascular space, pulse wave velocity, small vessel disease

## Abstract

**Objective:**

We investigated the effect of arterial stiffness on the severity of enlarged perivascular spaces (EPVSs) and cerebral microbleeds (CMBs) at different brain locations.

**Methods:**

A total of 854 stroke patients underwent both brachial‐ankle pulse wave velocity (baPWV) measurement and brain MRI. The extent of EPVS was separately rated at the levels of the basal ganglia (BG) and centrum semiovale (CS). The CMBs were categorized as strictly lobar CMB and deep CMB. The patients were categorized according to baPWV quartiles, and multivariable logistic regressions were performed to evaluate whether the baPWV increment was independently associated with each cerebral SVD marker at different locations. The odds ratio (OR) with 95% confidence interval (CI) was derived on the reference of the first quartile.

**Results:**

Severe EPVSs at BG and CS were detected in 243 (28.5%) and 353 patients (41.3%), respectively. The increment of baPWV quartiles was associated with both severe BG EPVS burden (Q4: OR = 2.58, CI = 1.45–4.60) and severe CS EPVS burden (Q4: OR = 2.06, CI = 1.24–3.42). Deep CMBs were found in 259 patients (30.3%), and strictly lobar CMBs were found in 170 patients (19.9%). Multivariable logistic regression model revealed deep CMB was independently associated with the baPWV increment (Q4: OR = 2.52, CI = 1.62–3.94). However, strictly lobar CMB had a neutral relationship with baPWV.

**Conclusion:**

Increased arterial stiffness is consistently associated with the presence of deep CMB and severe EPVS burden at the BG and CS, suggesting a common pathophysiologic mechanism.

## Graphical abstract

We investigated the effect of arterial stiffness on the severity of enlarged perivascular spaces (EPVSs) and cerebral microbleeds (CMBs) at different brain locations. A total of 854 stroke patients underwent both brachial‐ankle pulse wave velocity (baPWV) measurement and brain MRI. Increased arterial stiffness is consistently associated with the presence of deep CMB and severe EPVS burden at the BG and CS, suggesting a common pathophysiologic mechanism.

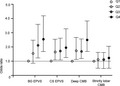

## 1 INTRODUCTION

Cerebral small vessel disease (SVD) refers to pathological processes that affect the arterioles, venules, and capillaries of the brain. The most common causes are aging, hypertension, and cerebral amyloid angiopathy (Pantoni, 2010). The main imaging features of SVD include lacunar infarction, hemorrhages, lacunes, white matter hyperintensities (WMHs), brain atrophy, cerebral microbleeds (CMBs), and enlarged perivascular spaces (EPVSs) (Wardlaw et al., 2013). Perivascular spaces are cerebrospinal fluid‐filled cavities that surround small penetrating cerebral arterioles. They are assumed to form a network of spaces around cerebral microvessels that act as a conduit for fluid transport, exchange between cerebrospinal fluid and interstitial fluid (Weller et al., 2009). Perivascular spaces are normally microscopic, but EPVSs become visible on T2‐weighted brain magnetic resonance imaging (MRI). EPVSs are one of the novel characteristic MRI features of SVD (Doubal et al., 2010; Pantankar et al., 2005; Potter, Doubal, et al., 2015).

Arterial stiffness exposes cerebral small vessels to abnormally high pulsatile pressure, inducing SVD (Poels et al., 2012). Previous studies have demonstrated a positive correlation between arterial stiffness and the various types of cerebral SVD markers, including CMB and EPVS (Kim et al., 2016; Poels et al., 2012; Riba‐Llena et al., 2018; Xiao et al., 2015; Zhai et al., 2018). The pathophysiology of EPVS and CMB in the deep brain structure and lobar areas is believed to differ (Greenberg et al., 2009; Pollock et al., 1997; Zhu et al., 2010). BG EPVS and deep CMB are typically associated with hypertensive vasculopathy (Greenberg et al., 2009; Zhai et al., 2018), whereas CS EPVS and lobar CMB are related to amyloid angiopathy (Greenberg et al., 2009; Martinez‐Ramirez et al., 2018). We investigated the association between baPWV increment and the location of EPVS and CMB in the deep brain structures and cerebral cortices of Korean stroke patients.

## 2 METHODS

### 2.1 Standard protocol approvals, registrations, and patient consents

This study was reviewed and approved by the institutional review boards at Chung‐Ang University Hospital.

### 2.2 Patient population

**FIGURE 1:**
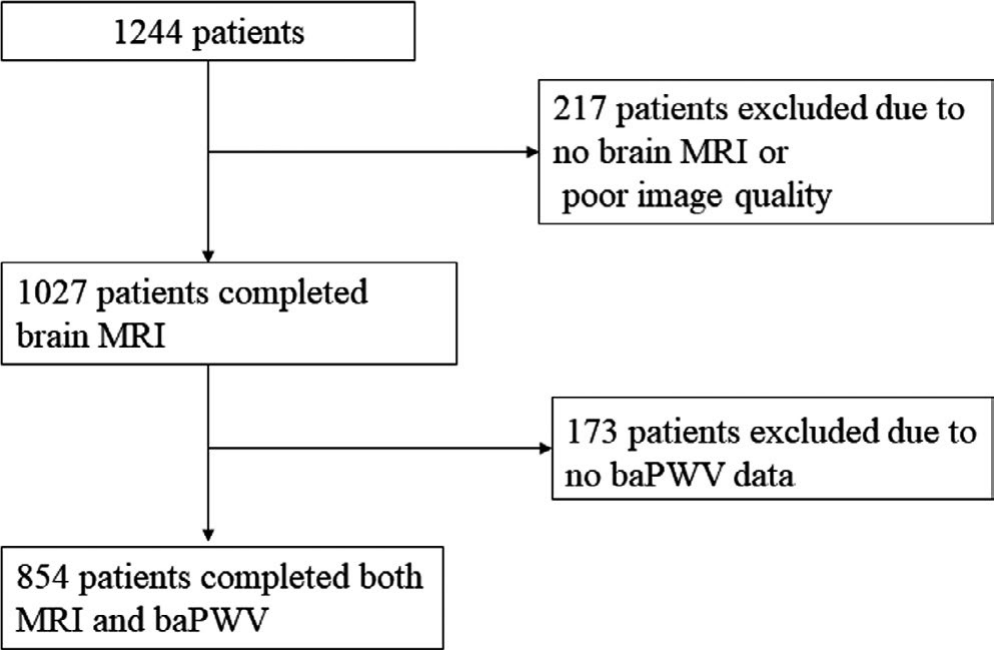
Flowchart of patient inclusion and exclusion in the study. baPWV, brachial‐ankle pulse wave velocity; MRI, magnetic resonance imaging

Between January 2014 and May 2017, 1,244 consecutive patients with acute ischemic stroke or transient ischemic attack who visited a tertiary university hospital in Seoul, Korea, within seven days of symptom onset were considered for inclusion. Among those, the 854 patients who had undergone both brain MRI and baPWV evaluation during admission were included (412 female patients, mean age = 68.2 ± 12.5 years) (Figure 1). Vascular risk factor profiles and laboratory data were obtained from the prospective stroke registry database. Hypertension was defined as blood pressure ≥ 140/90 mmHg measured on several separate occasions after the patient had been stabilized and started on antihypertensive medications. Diabetes mellitus was defined as receipt of antidiabetic medications before stroke or glycated hemoglobin A1c (HbA1c) exceeding 6.5%. Laboratory data included hematocrit, serum white blood cell count, platelet count, fasting blood sugar and HbA1c, cholesterol, high‐sensitivity C‐reactive protein (hsCRP), and estimated glomerular filtration rate (eGFR). eGFR was calculated by using modification of diet in the renal disease formula: eGFR (ml/min/1.73 m^2^) = 175 × (serum creatinine)^−1.154^ × age^−0.203^ × (0.742 if female).

### 2.3 Brachial‐ankle pulse wave velocity (baPWV)

The baPWV was measured with patients in the supine position using a volume‐plethysmography device (VP‐1000; Collin, Komaki, Japan) as reported previously (Tanaka et al., 2009). The device records the phonogram, volume pulse form, and arterial blood pressure at the brachia and ankles bilaterally. The baPWV was automatically calculated as the transmission distance divided by the transmission time. The distance between the right brachium and ankle was estimated on the basis of height. The average of the baPWV values obtained on both sides was used for further analysis.

### 2.4 Brain MRI protocol and the definition of cerebral small vessel disease markers

Brain MRI was performed with a 3.0‐T MR unit (Avanto, Philips, Eindhoven, The Netherlands), and the definitions were applied according to the standards for reporting vascular changes on neuroimaging (Wardlaw et al., 2013). EPVSs were defined as small, sharply demarcated structures of cerebrospinal fluid intensity on imaging measuring less than 3 mm that followed the pathway of the perforating vessels and ran perpendicular to the brain surface (Wardlaw et al., 2013). At the BG and CS levels, we estimated the slide in the most affected hemisphere. The numbers of EPVS were rated as follows: 0 = no EPVS, 1 = 1 to 10 EPVS, 2 = 11 to 20 EPVS, 3 = 21 to 40 EPVS, and 4 = more than 40 EPVS (Potter, Chappell, et al., 2015). CMBs were visualized as areas of homogenous low signals, either round or oval in shape and less than 10 mm in size on susceptibility‐weighted imaging (Wardlaw et al., 2013). CMBs were categorized as strictly lobar microbleeds or deep microbleeds based on their location. Strictly lobar CMBs were cortical and subcortical regions assessed in the frontal, parietal, temporal, occipital, and insular regions. Deep regions included basal ganglia, thalamus, internal capsule, external capsule, corpus callosum, and periventricular white matter (Gregoire et al., 2009). We categorized patients as either who had microbleeds restricted to a lobar location (strictly lobar microbleeds) or who had microbleeds in a deep location with or without lobar microbleeds (deep microbleeds). Lacunes were defined as round or ovoid, subcortical, fluid‐filled cavities between 3 mm and 15 mm in diameter, which generally had a central CSF‐like intensity on T1‐weighted and T2‐weighted MRI (Wardlaw et al., 2013). WMHs of vascular origin were hyperintense on T2‐weighted sequences (Wardlaw et al., 2013). The total cerebral small vessel disease (SVD) score on an ordinal scale from 0 to 4 was calculated based on a previously described scoring method that incorporates 4 SVD markers (Klarenbeek et al., 2013; Staals et al., 2014). Cerebral small vessel disease markers were rated by the two experienced neurologists (KJM and BJH) who were unaware of the clinical data, and inter‐rater testing showed good inter‐rater reliability with kappa values of 0.785 for EPVS in the basal ganglia, 0.794 for EPVS in the centrum semiovale, 0.805 for WMH, 0.856 for deep CMB, 0.835 for strictly lobar CMB, and 0.834 for lacune.

### 2.5 Statistical analyses

Statistical analysis was performed using Statistical Package for the Social Sciences version 18.0 (SPSS). First, we examined the relationship between clinical variables and baPWV. The patients were categorized into quartiles according to the baPWV, and demographic and laboratory variables of each quartile group were compared using analysis of variance (ANOVA) testing with the post hoc Bonferroni test or linear‐by‐linear association. Next, to determine whether the effect of arterial stiffness differs according to the anatomic location of the EPVS, we dichotomized the study population according to the EPVS burden at BG and CS into mild (score of 1–2) and severe (score of 3–4) using the previous definitions (Potter, Chappell, et al., 2015). We also dichotomized the study population according to the presence of deep or strictly lobar CMB. Demographic and laboratory information was compared by bivariate analysis with the independent *t* test or chi‐square test. Multivariable logistic regression analyses were performed by including the variables derived from the bivariate analysis and with biological relevance. With the first quartile as a reference, the odds ratios (OR) of each quartile were derived for cerebral SVD burden with 95% confidence interval (CI).

## 3 RESULTS

Among the 854 patients, 243 patients (28.5%) had severe BG EPVS and 353 patients (41.3%) had severe CS EPVS; 259 patients (30.3%) had deep CMB, and 170 (19.9%) had strictly lobar CMB. The median value of the total SVD score was 2.0, and the mean baPWV was 20.6 ± 5.6 m/s. The higher baPWV quartile was proportionally associated with old age, female sex, hypertension, diabetes mellitus, previous stroke history, blood pressure, fasting blood sugar, and hsCRP. The proportion of current smokers, estimated glomerular filtration rate, and hematocrit level were inversely related to the baPWV quartile. The extent and severity of each cerebral SVD phenotype tended to increase according to the baPWV quartile, except for strictly lobar CMB (Table 1). The presence of strictly lobar CMB remained relatively stable at approximately 20% throughout each baPWV quartile.

**TABLE 1:** Comparison of clinical variables according to the quartiles of mean brachial‐ankle pulse wave velocities

	Q1	Q2	Q3	Q4	*p*
Patient numbers	213	214	213	214	
Age, years (*SD*)	57.8 (12.6)	67.4 (10.2)	70.8 (10.3)	76.8 (8.1)	<.001
Sex, female, *n* (%)	84 (39.4)	92 (43.0)	93 (43.7)	143 (66.8)	<.001
BMI, kg/m^2^, mean (*SD*)	24.3(3.6)	24.3 (3.3)	24.2 (3.0)	24.3 (13.3)	.961
Hypertension, *n* (%)	82 (38.4)	134 (62.6)	151 (70.9)	163 (76.2)	<.001
Diabetes mellitus, *n* (%)	33 (15.4)	62 (29.0)	78 (36.6)	85 (39.7)	<.001
Smoking, *n* (%)	72 (33.8)	62 (29.0)	61 (28.6)	42 (19.6)	<.001
Atrial fibrillation, *n* (%)	37 (17.3)	39 (18.2)	42 (19.7)	18 (8.4)	.490
Previous stroke, *n* (%)	33 (15.4)	36 (16.8)	49 (23.0)	46 (21.5)	.045
SBP, mm/Hg, mean (*SD*)	139 (24)	149 (26)	154 (28)	157 (29)	<.001
DBP, mm/Hg, mean (*SD*)	83 (13)	87 (15)	86 (16)	88 (14)	.004
Hematocrit, %, mean (*SD*)	41.4 (5.0)	40.7 (7.0)	40.4 (5.6)	39.7 (4.8)	.002
White blood cell, 10^9^/L, mean (*SD*)	8.0 (3.1)	8.1 (3.2)	8.2 (2.9)	8.0 (3.3)	.741
Platelet, 10^9^/L, mean (*SD*) (*SD*)	230.0 (53.6)	236.4 (80.2)	235.8 (70.3)	236.8 (85.3)	.405
Fasting blood sugar, mmol/L, mean (*SD*)	7.3 (3.4)	7.5 (2.3)	8.2 (3.2)	8.2 (3.6)	<.001
LDL cholesterol, mmol/L, mean (*SD*)	2.7 (0.8)	2.8 (0.8)	2.7 (0.8)	2.7 (0.9)	.923
Triacylglycerol, mmol/L, mean (*SD*)	3.0 (2.3)	3.0 (1.8)	3.3 (2.1)	3.0 (1.7)	.806
hsCRP, mg/dl, mean (*SD*)	4.1(19.4)	5.2(13.5)	6.3(18.4)	8.9(24.3)	.009
eGFR, ml/min/1.73 m^2^, mean (*SD*)	90.2 (23.0)	82.5 (25.8)	77.3 (25.8)	76.5 (27.4)	<.001
Severe WMH, *n* (%)	7 (3.3)	24 (11.2)	23 (10.8)	47 (22.0)	<.001
Lacune, *n* (%)	100 (46.9)	138 (64.5)	157 (73.7)	183 (85.5)	<.001
Deep CMB, *n* (%)	41 (19.2)	65 (30.4)	67 (31.5)	86 (40.2)	<.001
Strictly lobar CMB, *n* (%)	35 (16.4)	43 (20.1)	44 (20.7)	48 (22.4)	.129
BG PVS, severe, *n* (%)	23 (10.8)	51 (23.8)	70 (32.9)	99 (46.3)	<.001
CS PVS, severe, *n* (%)	43 (20.2)	87 (40.7)	99 (46.5)	124 (57.9)	<.001
Total SVD score, median (*SD*)	1.2 (1.0)	1.93 (1.2)	2.1 (1.0)	2.6 (0.9)	<.001

Note

The cutoff values of mean baPWV for each quartile were 1,663, 1,985, and 2,355 cm/s. *p* Value was derived from analysis of variance.

Abbreviations: BG, basal ganglia; BMI, body mass index; CMB, cerebral microbleed; CS, centrum semiovale; DBP, diastolic blood pressure; eGFR, estimated glomerular filtration rate; hsCRP, high‐sensitive C‐reactive protein; LDL, low‐density lipoprotein; PVS, perivascular space, SBP, systolic blood pressure; *SD*, standard deviation; SVD, small vessel disease; WMH, white matter hyperintensities.

**FIGURE 2:**
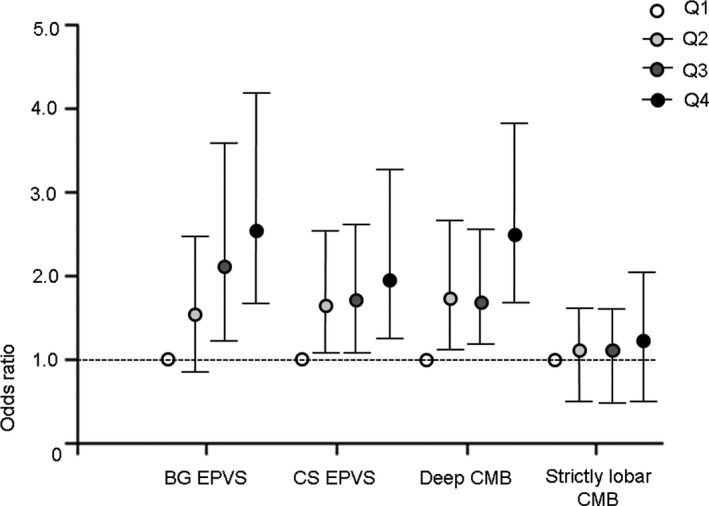
Relationship between arterial stiffness and cerebral small vessel disease markers at different locations. When the patients were grouped according to quartiles of brachial‐ankle pulse wave velocity, enlarged perivascular spaces (EPVS) at the basal ganglia and centrum semiovale and deep CMB showed similar patterns of increase. BG EPVS (OR per baPWV quartile, Q2: 1.63, 95% CI 0.92–2.88; Q3: 2.11, 1.20–3.71; and Q4: 2.58, 1.45–4.60). CS EPVS (OR per baPWV quartile, Q2: 1.71, 1.07–2.73; Q3: 1.72, 1.06–2.78; and Q4: 2.06, 1.24–3.42). Deep CMB (OR per baPWV quartile, Q2: 1.74, 1.10–2.73; Q3: 1.70, 1.08–2.69; and Q4: 2.52, 1.62–3.94). Strictly lobar CMB (OR per baPWV quartile, Q2: 1.13, 0.69–1.85; Q3: 1.14, 0.68–1.93; and Q4: 1.30, 0.73–2.30). The cutoff values of mean brachial‐ankle pulse wave velocity for each quartile were 1,663, 1,985, and 2,355 cm/s. BG, basal ganglia; CMB, cerebral microbleed; CS, centrum semiovale; PVS, perivascular space

When the patients were dichotomized according to the EPVS burden, severe BG EPVS and CS EPVS both were proportionally associated with age, female sex, hypertension, previous stroke history, hematocrit, hsCRP, lower eGFR, and mean baPWV (Table 2). Higher baPWV quartile was associated with both BG EPVS (OR per baPWV quartile, Q2: OR 1.63, 95% CI 0.92–2.88; Q3: 2.11, 1.20–3.71; and Q4: 2.58, 1.45–4.60) and CS EPVS (OR per baPWV quartile, Q2: 1.71, 1.07–2.73; Q3: 1.72, 1.06–2.78; and Q4: 2.06, 1.24–3.42) after adjusting for age, female sex, hypertension, smoking, previous stroke history, hematocrit, platelet, triacylglycerol, hsCRP, and eGFR (Figure 2).

**TABLE 2:** Comparison of clinical variables according to the location of EPVS

	Basal ganglia			Centrum semiovale		
	None–moderate *n* = 611	Severe *n* = 243	*p*	None–moderate *n* = 501	Severe *n* = 353	*p*
Age, years, mean (*SD*)	65.5 (12.7)	75.0 (8.6)	<.001	64.6 (13.1)	73.3 (9.5)	<.001
Sex, female, *n* (%)	270 (44.2)	142 (58.4)	<.001	226 (45.1)	186 (52.7)	.029
BMI, kg/m^2^, mean (*SD*)	24.5 (8.3)	23.8 (3.5)	.228	24.2 (3.3)	24.4 (10.5)	.741
Hypertension, *n* (%)	351 (57.4)	179 (73.7)	<.001	273 (54.5)	257 (72.8)	<.001
Diabetes mellitus, *n* (%)	184 (30.1)	74 (30.5)	.923	151 (30.1)	107 (30.3)	.957
Smoking, *n* (%)	185 (30.3)	28 (11.5)	<.001	149 (29.7)	64 (18.1)	<.001
Atrial fibrillation, *n* (%)	117 (19.1)	43 (17.7)	.623	98 (19.6)	62 (17.6)	.461
Previous stroke, *n* (%)	94 (15.4)	70 (28.8)	<.001	71 (14.2)	93 (26.3)	<.001
SBP, mm/Hg, mean (*SD*)	149 (27)	152 (30)	.181	149 (28)	152 (27)	.127
DBP, mm/Hg, mean (*SD*)	86 (15)	86 (15)	.480	86 (15)	86 (14)	.889
Hematocrit, %, mean (*SD*)	41.0 (5.4)	39.4 (6.3)	<.001	41.0 (5.4)	39.9 (6.0)	.005
White blood cell, 10^9^/L, mean (*SD*)	8.1 (3.0)	8.1 (3.5)	.994	8.0 (2.7)	8.2 (3.6)	.242
Platelet, 10^9^/L, mean (*SD*)	231.4 (65.3)	243.5 (89.8)	.029	234.6 (64.0)	235.1 (84.9)	.929
Fasting blood sugar, mmol/L, mean (*SD*)	7.8 (3.3)	7.6 (3.0)	.430	7.8 (3.2)	7.7 (3.2)	.629
LDL cholesterol, mmol/L, mean (*SD*)	2.7 (0.8)	2.7 (0.9)	.546	2.8 (0.8)	2.7 (0.8)	.408
Triacylglycerol, mmol/L, mean (*SD*)	3.2 (2.1)	2.8 (1.7)	.027	3.1 (2.0)	3.0 (2.0)	.538
hsCRP, mg/dl, mean (*SD*)	5.1 (16.4)	8.8 (25.2)	.011	4.2 (11.2)	8.9 (26.8)	<.001
eGFR, ml/min/1.73m^2^, mean (*SD*)	83.4 (25.7)	77.1 (26.7)	.001	84.2 (25.4)	77.9 (26.6)	<.001
Mean baPWV, m/s, mean (*SD*)	2.0 (0.5)	2.3 (0.6)	<.001	1.9 (0.5)	2.2 (5.7)	<.001

Abbreviations: baPWV, brachial‐angle pulse wave velocity; BMI, body mass index; DBP, diastolic blood pressure; eGFR, estimated glomerular filtration rate; hsCRP, high‐sensitive C‐reactive protein; LDL, low‐density lipoprotein, SBP, systolic blood pressure; *SD*, standard deviation.

Deep CMB was associated with age, hypertension, smoking, previous stroke history, higher blood pressure, lower eGFR, and higher mean baPWV. However, strictly lobar CMB showed an association only with older age (Table 3). Higher baPWV quartile was proportionally associated with deep CMB (OR per baPWV quartile, Q2 1.74, 1.10–2.73; Q3: 1.70, 1.08–2.69; and Q4: 2.52, 1.62–3.94) after adjusting for age, hypertension, smoking, previous stroke history, higher blood pressure, and eGFR (Figure 2).

**TABLE 3:** Comparison of clinical variables according to the location of CMB

	Deep CMB			Strictly lobar CMB		
	CMB (−) *N* = 595	CMB (+) *N* = 259	*p*	CMB (−) *N* = 684	CMB (+) *N* = 170	*p*
Age, years, mean (*SD*)	67.4 (12.7)	70.2 (11.6)	.002	67.8 (12.6)	70.1 (11.8)	.028
Sex, female, *n* (%)	276 (46.4)	136 (52.5)	.100	328 (48.0)	84 (49.4)	.733
BMI, kg/m^2^, mean (*SD*)	24.1 (3.2)	24.7 (12.2)	.292	24.4 (7.9)	24.0 (3.4)	.539
Hypertension, *n* (%)	355 (59.7)	175 (67.6)	.029	419 (61.3)	111 (65.3)	.332
Diabetes mellitus, *n* (%)	180 (30.3)	78 (30.1)	.968	207 (30.3)	51 (30.0)	.947
Smoking, *n* (%)	161 (27.1)	52 (20.1)	.030	167 (24.4)	46 (27.1)	.476
Atrial fibrillation, *n* (%)	107 (18.0)	53 (20.5)	.393	126 (18.4)	34 (20.0)	.637
Previous stroke, *n* (%)	96 (16.1)	68 (26.3)	.001	127 (18.6)	37 (21.8)	.344
SBP, mm/Hg, mean (*SD*)	148 (26)	155 (31)	.001	150 (28)	149 (27)	.819
DBP, mm/Hg, mean (*SD*)	85 (14)	88 (16)	.008	86 (15)	86 (16)	.886
Hematocrit, %, mean (*SD*)	40.6 (5.2)	40.5 (6.8)	.747	40.7 (5.7)	39.8 (5.7)	.060
White blood cell, 10^9^/L, mean (*SD*)	8.1 (3.0)	8.1(3.4)	.780	8.1 (3.2)	8.1 (2.8)	.847
Platelet, 10^9^/L, mean (*SD*)	233.7 (67.8)	237.5 (84.6)	.478	235.1 (74.2)	233.9 (69.8)	.849
Fasting blood sugar, mmol/L, mean (*SD*)	7.8 (3.2)	7.7 (3.2)	.596	7.8 (3.3)	7.7 (2.7)	.842
LDL cholesterol, mmol/L, mean (*SD*)	2.7 (0.8)	2.7 (0.9)	.531	2.7 (0.8)	2.7 (0.9)	.551
Triacylglycerol, mmol/L, mean (*SD*)	3.1 (2.1)	3.0 (1.8)	.209	3.1 (2.0)	3.2 (2.2)	.391
hsCRP, mg/dl, mean (*SD*)	5.6 (16.1)	7.3 (25.4)	.236	6.2 (20.0)	5.8 (16.5)	.820
eGFR, ml/min/1.73 m^2^, mean (*SD*)	83.7 (25.1)	77.0 (27.6)	.001	81.2 (26.2)	83.3 (25.5)	.339
Mean baPWV, m/s, mean (*SD*)	2.0 (0.5)	2.2 (0.6)	<.001	2.0 (0.6)	2.1 (0.5)	.168

Abbreviations: baPWV, brachial‐angle pulse wave velocity; BMI, body mass index; DBP, diastolic blood pressure; eGFR, estimated glomerular filtration rate; hsCRP, high‐sensitive C‐reactive protein; LDL, low‐density lipoprotein; SBP, systolic blood pressure; *SD*, standard deviation.

## 4 DISCUSSION

This study provides clues about the pathophysiologic mechanisms underlying cerebral SVD phenotypes by illustrating the relationship between arterial stiffness and SVD burden at different brain locations. The burdens of EPVS at the BG and CS levels were consistently related to conventional vascular risk factors and independently associated with increased baPWV. Although the presence of deep CMB was also related to increased baPWV, the strictly lobar CMB did not show any relationship with baPWV, suggesting a different pathophysiologic mechanism beyond hypertensive arteriopathy.

Elastic components of the arterial wall decrease because of aging processes, hypertension, atherosclerosis, and vascular wall calcification, leading to increased pulse wave velocity (Lee & Oh, 2010). Increased arterial stiffness is responsible for a disproportionate increase in systolic arterial pressure and decrease in diastolic pressure, which results in circumferential stretching of the arterial wall, leading to intimal fibrosis, necrosis, remodeling, and atherosclerosis of the vessels (Mitchell et al., 2011). These changes lead to the transmission of higher pulse pressure to distal organs (Laurent et al., 2009). The brain is a distal organ with a large amount of blood flow, and it is particularly susceptible to excessive pulsatile stress because of low cerebral vascular resistance (Mitchell et al., 2011). Increased arterial pulsatility can drive glymphatic influx to the brain parenchyma and blood–brain barrier break down, which causes greater extravasation of fluid into the perivascular space (Kress et al., 2014; Mestre et al., 2017). Multiple studies have consistently shown the association between increased arterial PWV and increased burden of cerebral SVD such as WMH, CMBs, lacunes, and EPVS (Kim et al., 2016; Poels et al., 2012; Riba‐Llena et al., 2018; Zhai et al., 2018).

The pathophysiology of CS EPVS and lobar CMB has been considered to be related to amyloid angiopathy based on analysis of small numbers of postmortem brain specimen (Greenberg et al., 2009; Martinez‐Ramirez et al., 2018). However, more recent studies have reported consistent associations of both BG and CS EPVS with hypertensive arteriopathy, suggesting that overlapping pathophysiology of hypertensive vasculopathy and amyloid angiopathy contribute to CS EPVS (Shams et al., 2017; Yakushiji et al., 2014). Our study results also show that BG EPVS, CS EPVS, and deep CMB are closely related to increased baPWV, sharing arterial stiffness as a main mechanism of injury. However, strictly lobar CMB seemed to be independent of hypertensive vasculopathy, which accords with previous findings.

This study has several limitations. First, some patients who did not undergo brain MRI or baPWV measurement were excluded from the study, which may have caused selection bias. Second, the study was based in a single center and included an ethnically homogeneous population. A causal relationship between baPWV and EPVS could not be ascertained because of the cross‐sectional design. Finally, increased baPWV might be a consequence of decreased physical activity due to cerebral SVD burden.

In conclusion, this study shows that increased baPWV, which is regarded as a marker of arterial stiffness, has an independent association with the presence of EPVS and deep CMB at both the BG and CS, but has a neutral relationship with strictly lobar CMB. This consistent relationship between EPVS at both levels and arterial stiffness suggests a common pathological mechanism related to hypertensive SVD damage.

## CONFLICT OF INTEREST

Jae‐Han Bae, MD, reports no disclosures. Jeong‐Min Kim, MD, PhD, is funded by the Basic Science Research Program through the National Research Foundation of Korea (NRF), the Ministry of Education in Republic of Korea (NRF‐2019R1F1A1059455). Kwang‐Yeol Park, MD, PhD, is funded by the Basic Science Research Program through the National Research Foundation of Korea (NRF), the Ministry of Education in Republic of Korea (NRF‐2017R1D1A1B03029909). Su‐Hyun Han, MD, reports no disclosures.

## AUTHOR'S CONTRIBUTIONS

Kwang‐Yeol Park and Jeong‐Min Kim participated in the conception and design of the study. Jae‐Han Bae and Su‐Hyun Han collected the data. Kwang‐Yeol Park and Jeong‐Min Kim analyzed the data. Jeong‐Min Kim and Jae‐Han Bae wrote the manuscript.

### Peer Review

The peer review history for this article is available at https://publons.com/publon/10.1002/brb3.1935.

## Data Availability

Anonymized data can be shared at the request of qualified investigators for purposes of replicating procedures and results.
